# Improving the thermostability and stress tolerance of an archaeon hyperthermophilic superoxide dismutase by fusion with a unique N-terminal domain

**DOI:** 10.1186/s40064-016-1854-9

**Published:** 2016-03-01

**Authors:** Mingchang Li, Lin Zhu, Wei Wang

**Affiliations:** Key Laboratory of Molecular Microbiology and Technology, Ministry of Education, TEDA Institute of Biological Sciences and Biotechnology, Nankai University, 23 Hongda Street, TEDA, Tianjin, 300457 People’s Republic of China; Tianjin Key Laboratory of Microbial Functional Genomics, TEDA, Tianjin, 300457 People’s Republic of China

**Keywords:** Superoxide dismutase, Thermostability, Stress tolerance, Bioengineering, *Geobacillus thermodenitrificans* NG80-2, *Sulfolobus solfataricus*

## Abstract

The superoxide dismutase from the archaeon *Sulfolobus solfataricus* (SOD_*Ss*_) is a well-studied hyperthermophilic SOD with crystal structure and possible thermostability factors characterized. Previously, we discovered an N-terminal domain (NTD) in a thermophilic SOD from *Geobacillus thermodenitrificans* NG80-2 which confers heat resistance on homologous mesophilic SODs. The present study therefore aimed to further improve the thermostability and stress tolerance of SOD_*Ss*_ via fusion with this NTD. The recombinant protein, rSOD_*Ss*_, exhibited improved thermophilicity, higher working temperature, improved thermostability, broader pH stability, and enhanced tolerance to inhibitors and organic media than SOD_*Ss*_ without any alterations in its oligomerization state. These results suggest that the NTD is an excellent candidate for improving stability of both mesophilic and thermophilic SOD from either bacteria or archaea via simple genetic manipulation. Therefore, this study provides a general, feasible and highly useful strategy for generating extremely thermostable SODs for industrial applications.

**Electronic supplementary material:**

The online version of this article (doi:10.1186/s40064-016-1854-9) contains supplementary material, which is available to authorized users.

## Background

Superoxide dismutases (SODs, EC 1.15.1.1), which is one of the most important metalloenzymes in the first line of defense against oxidative stress, catalyze the dismutation of the superoxide anion (O^2−^) into hydrogen peroxide and molecular oxygen (Fridovich 1978; Imlay 2008). Four different types of metal centers have been detected in SODs, dividing this family into Cu/Zn-, Mn-, Fe- and Ni-SODs (Miller 2012). Of these, Cu, Zn-SODs, and probably Ni-SODs, are structurally distinct from Fe- and Mn-SODs which consist of dimers or tetramers that share substantial sequence similarity and possess virtually identical protein folds and active-site geometries (Jackson and Brunold 2004). A few cambialistic SODs, however, can fulfill their function with both Fe^2+^ and Mn^2+^ as cofactors (Edward et al. 1998).

SODs are widely used in cosmetics, health care products, agriculture as well as pharmaceuticals due to their generally vast bioavailability, high affinity and elimination rates with reactive oxygen species (ROS) (Bafana et al. 2011). For industrial applications, it is preferable that an enzyme has both structural and functional stability under severe conditions. The thermostability is one of the most important properties that have been discussed since thermal denaturation is a common cause of enzyme inactivation in industry (Wang et al. 2008b). Moreover, better thermostability is always associated with a higher tolerance to chemical denaturants (Vieille and Zeikus 2001). To date, many thermostable SODs have been reported and characterized from thermophiles and hyperthermophiles, such as the Fe-SODs from *Rhodothermus* sp. (Wang et al. 2008b) and *Aquifex pyrophilus* (Lim et al. 1997), the Mn-SODs from *Thermus thermophiles* (Zhu et al. 2013) and *Chaetomium thermophilum* (Haikarainen et al. 2014), and the cambialistic SODs from *Pyrobaculum calidifontis* (Amo et al. 2003) and *Propionibacterium shermanii* (Meier et al. 1997).

Recent efforts to improve the thermostability and stress tolerance of SODs through enzyme immobilization (Song et al. 2012), chemical modification (Zhang et al. 2006), mutagenesis of specific amino acids (Kumar et al. 2012), SOD mimics (Pinto et al. 2013) and combination with chaperone proteins or other agents (Bresson-rival 1999) have achieved considerable success. However, it is extremely difficult to bioengineer a specific enzyme with enhanced thermostable with a “universal” method, since the determinants of enzyme thermostability are numerous, including factors such as amino acid composition, disulfide bridges, aromatic interactions, hydrophobic effect, hydrogen bonds, ion pairs, intersubunit interactions, nonlocal versus local interactions, helix dipole stabilization, posttranslational modifications, packing efficiency, conformational strain release, anchoring of loose ends, docking of the N or C termini, extrinsic parameters, and metal binding (Vieille and Zeikus 2001). Optimising the structural stability of a SOD, especially a thermophilic SOD, faces great challenges.

In the previous work we have discovered a unique 244-amino acid N-terminal domain (NTD) that confers heat resistance to the Fe/Mn-SOD_*NG2215*_ of *Geobacillus thermodenitrificans* NG80-2, a crude oil-degrading thermophilic facultative anaerobe (Wang et al. 2014b; Feng et al. 2007). A homologous mesophilic SODA_*BSn5*_ was evolved to a moderately thermophilic enzyme by fusion with NTD of SOD_*NG2215*_, providing new clues for improving thermostability of mesophilic SOD. However, whether and how this strategy will affect the natural thermophilic SODs becomes a more interesting question. One of the most studied thermophilic and thermostable SODs, Fe-SOD_*Ss*_ from the hyperthermophilic archaeon *Sulfolobus solfataricus* (Brock et al. 1972), was well determined of crystal structure and analysed of thermostability factors (Yamano and Maruyama 1999; Ursby et al. 1999; Dello Russo et al. 1997). Thus, SOD_*Ss*_ provides us a specific object to study the effect of NTD to the natively thermostable enzyme.

In this study, we recombined the NTD to the N-terminal of SOD_*Ss*_ to further modify natively thermostable SOD. The biochemical properties (e.g. optimum temperature and pH, thermal stability, acidic and alkaline stability,stress stability) of the fusion protein (rSOD_*Ss*_) were characterized and compared with those of SOD_*Ss*_. In addition, the possible mechanisms responsible for improvement in enzyme stability were explored through analysis of oligomerization state and comparison of structural modelling. The work presented here may provide a general and feasible strategy to enhance the thermophilicity and tolerance of both mesophilic and thermophilic Fe- or Mn-SODs from either bacteria or archaea.

## Methods

### Cloning and plasmid construction

Gene of SOD_*Ss*_ (GenBank accession number: AB012620.1) from *Sulfolobus solfataricus* was synthesised into pET-28a by GENEWIZ Biological Technology Co., Ltd. (Beijing, China), thus generating pET-SOD_*Ss*_. Genomic DNA (GenBank: CP000557.1) from NG80-2 was extracted as previously described (Feng et al. 2007). The primers used in this study are listed in Additional file 1: Table S1. The PCR was initiated by denaturation at 95 °C for 3 min, followed by 30 cycles of 95 °C for 30 s, 55 °C for 45 s and 72 °C for 2 min 30 s and a final extension at 72 °C for 5 min. The sequence encoding the SOD NTD (*sod*_*GTNG_2215*-*N*_) was PCR-amplified using NG80-2 genomic DNA as the template. The active sequence of SOD_*Ss*_ (*sod*_*Ss*-*C*_) was obtained using pET-SOD_*Ss*_ as the template. The two fragments were used as a template to amplify the SOD-fusion enzyme sequence *rsod*_*Ss*_, which was then digested with EcoRI and HindIII and ligated into pET-28a digested with the same enzymes, generating pET-rSOD_*Ss*_. The presence of the insert in the recombinant plasmid was confirmed by sequencing using an ABI 3730 automated DNA sequencer (ABI, Foster City, CA, USA).

### Protein expression and purification

The pET-SOD_*Ss*_ and pET-rSOD_*Ss*_ were transformed into *E. coli* BL21 (DE3) for protein expression, which were grown in Luria–Bertani medium supplemented with kanamycin (50 μg ml^−1^) at 37 °C to an A600 nm of 0.6 and induced with 0.2 mM IPTG at 30 °C for 5 h. The cells were harvested by centrifugation and resuspended in lysis buffer (50 mM Tris–HCl, pH 8.0, 300 mM NaCl and 10 mM imidazole), and then disrupted by sonication (Hielscher UP200s ultrasonic processor, Teltow, Germany). Cell debris was removed by centrifugation at 12,000×*g* for 20 min. The crude extract was applied to a Chelating Sepharose Fast Flow column (GE Helthcare, Uppsala, Sweden) according to the manufacturer’s instructions. The eluted proteins were dialysed against 50 mM Tris–HCl (pH 8.0) containing 20 % glycerol.

The protein concentration was estimated by Bradford method (Bradford 1976). Sodium dodecyl sulphate polyacrylamide gel electrophoresis (SDS-PAGE) was performed according to the method described by Laemmli (1970).

### SOD activity assay

SOD activity was measured using the method of Beauchamp and Fridovich (Beauchamp and Fridovich 1971). Briefly, the 3-ml reaction mixture contained 13 mM l-methionine, 63 μM nitroblue tetrazolium (NBT), 1.3 μM riboflavin, 10 μM EDTA-Na_2_, and 10 μl purified enzyme in 50 mM potassium phosphate buffer (pH 7.8). The test tubes were exposed to a source of light at 25 °C. The reduction of NBT was monitored after 15 min at 560 nm. One unit of SOD activity was defined as the amount of enzyme that caused 50 % of maximum inhibition of the NBT reduction. All assays were performed in triplicate, and average values were reported. Activity was estimated as a percentage of the maximum.

### Effects of temperature and pH on SOD activity

To determine the optimum temperature, SOD activity was measured in the standard reaction mixture at temperatures ranging from 20 to 100 °C. To determine the optimum pH, SOD activity was measured in the pH range of 3.0–10.0 using 50 mM sodium citrate (pH 3.0–8.0), Tris–HCl (pH 8.0 and 9.0), or glycine-NaOH (pH 9.0 and 10.0) buffers. Activity was calculated as the percentage of the maximum. The biphasic deactivation nature of enzymes, including parameters of *k*_*d*_, *D*-value, *t*_*1/2*_ and *Ed*, were determined as described previously (Whittaker 1994; Belitz et al. 1999; Henley and Sadana 1985).

### Stability test

For thermostability testing, the native enzymes were incubated at 90, 95, 100 and 105 °C for 1–5 h without substrate. At various times, aliquots were taken and chilled on ice immediately. Subsequently, the residual activity was measured in assay buffer under the standard condition (pH 7.8, 25 °C) and calculated as the percentage of the maximum activity. The pH stability of SODs was determined by keeping the enzyme in buffers with different pH values (ranging from 3 to 10) at 25 °C for 90 min, followed by measuring residual activity under the standard assay condition.

### Effects of inhibitors, denaturants, detergents and organic medium on SOD activity

The effects of inhibitors, denaturants and detergents on SOD activity were determined by using ethylenediaminetetraacetic acid (EDTA) and β-mercaptoethanol (β-ME) at final concentrations of 1 or 10 mM, urea and guanidine hydrochloride at final concentrations of 2.5 M, sodium dodecyl sulfate (SDS) at final concentrations of 0.1 % (w/v or v/v) or 1 % (w/v or v/v). The enzyme was incubated with each inhibitor, denaturant and detergent at 25 °C for 30 min in 50 mM sodium phosphate buffer (pH 7.8), individually (Zhu et al. 2014). To test the stability of SODs in an organic medium, each enzyme was incubated in a 50 mM HEPES–KOH (pH 7.0) buffer supplemented with ethanol and ethylene glycol at final concentrations of 20 or 50 % at 25 °C for 30 min. Residual activities were measured by the standard assay as described above. Reaction mixture without additives was used as a reference (Nakamura et al. 2011).

### Analytical ultracentrifugation

Sedimentation velocity experiments were performed in a Proteome Lab XL-1 Protein Characterization System (Beckman Coulter). All interference data were collected at a speed of 36,000 rpm in an An-60 Ti rotor at 4 °C. A set of 200 scans was collected at 6-min intervals. The proteins were prepared in 50 mM potassium phosphate buffer plus 150 mM NaCl at pH 8.0. The data were analysed using the program SEDFIT (version 11.8) in terms of a continuous c(s) distribution (Wang et al. 2014b).

### Fitting to the equilibrium model

Reaction-progress curves at a variety of temperatures were determined. With *ΔG*_cat_^*‡*^ (80 kJ mol^−1^), *ΔG*_inact_^*‡*^ (95 kJ mol^−1^), *H*_eq_ (100 kJ mol^−1^) and *T*_eq_ (320 K) values as initial parameter estimates and enzyme concentration (mol l^−1^) in each assay, the experimental data were fitted to the equilibrium model using a stand-alone Matlab 7.1.0.246 (R14) (http://hdl.handle.net/10289/3791) as previously described (Peterson et al. 2007).

## Results

### Gene manipulation and construction of the expression plasmid

**Fig. 1:**
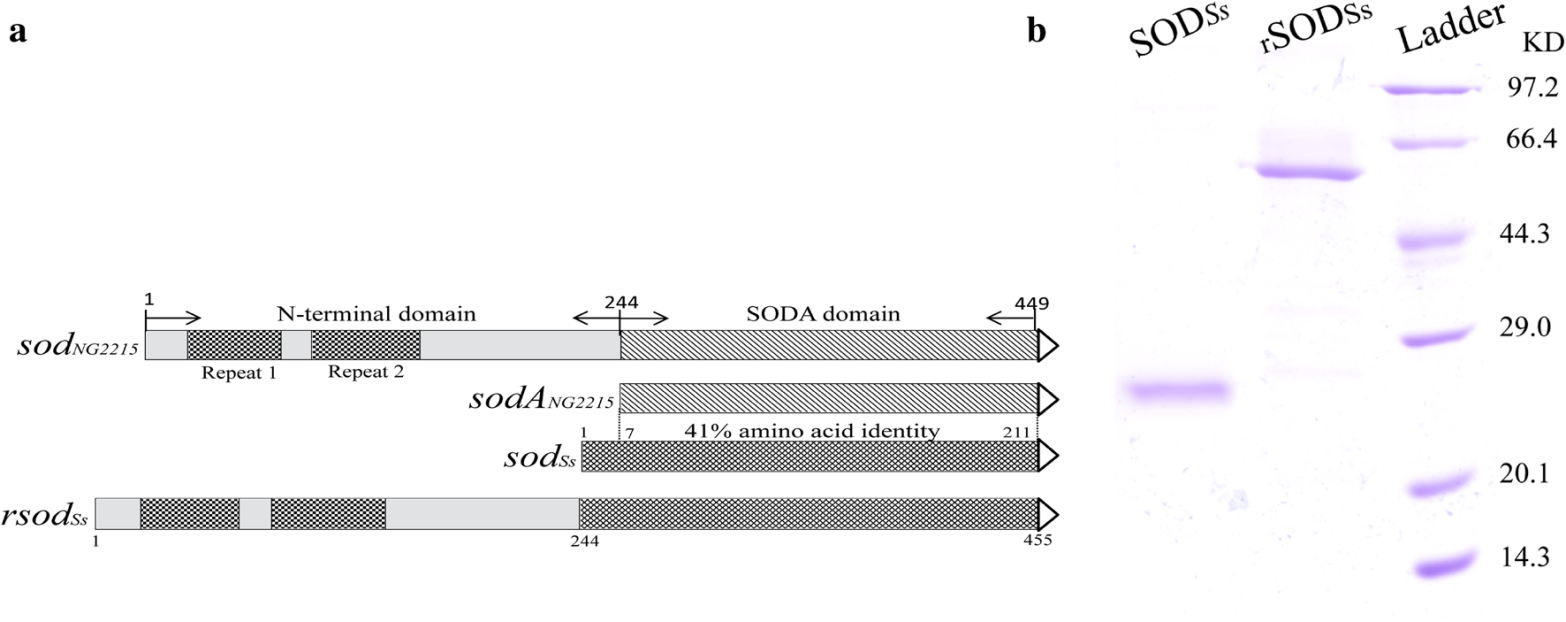
Schematic illustration of rSOD_*Ss*_ construction (**a**) and SDS-PAGE analysis of purified SOD_*Ss*_ and rSOD_*Ss*_ (**b**). Proteins were stained with Coomassie brilliant blue R-250. Ladder, standard protein size marker. The expected sizes of SOD_*Ss*_ and rSOD_*Ss*_ were 24.2 and 51.6 kDa, respectively

Two combinant clones have been constructed to comparatively study the thermostability and stress resistance of the SOD_*Ss*_ and rSOD_*Ss*_ with appendant NTD (Fig. 1a). Expression plasmid pET-rSOD_*Ss*_ was confirmed by DNA sequencing. In order to purify the recombinant proteins with Ni-NTA His·Bind Resin affinity chromatography, the cloned SOD_*Ss*_ and rSOD_*Ss*_ were fused with 6× histidine tag at N-terminus.

### Expression and purification of SOD variants

PET-SOD_*Ss*_ and pET-rSOD_*Ss*_ were transformed into *E. coli* BL21 (DE3) separately. After induction and lysis, the crude supernatant was applied onto the Ni-NTA His·Bind affinity chromatography for SODs purification. Purified proteins were subjected to electrophoresis on 12 % SDS-PAGE and the rough sizes of SOD_*Ss*_ and rSOD_*Ss*_ subunits observed were 24 and 51 kDa respectively, coinciding with the molecular masses calculated from the amino acid sequences derived from the genes (Fig. 1b).

### The NTD contributes to host thermophilicity with no alteration in its pH optimum

**Fig. 2:**
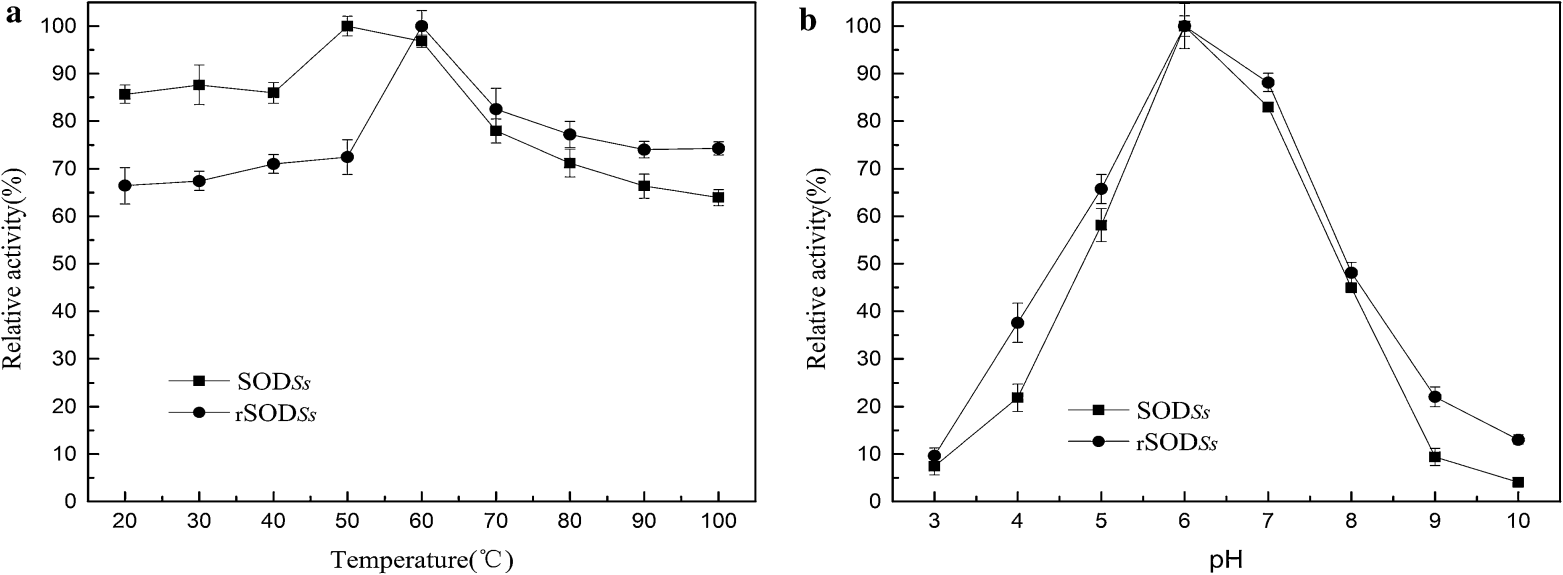
Effects of temperature (**a**) and pH (**b**) on SOD activity. The optimal temperatures were determined by assaying the activity of purified SOD at temperatures ranging from 20 to 100 °C, and the optimal pH values were determined in buffers ranging from pH 3–10. The activity at the optimal temperature or pH was defined as 100 %. Each point represents the mean (n = 3) ± the standard deviation

The optimum active temperature (OAT) was determined by testing the SOD activity at temperatures ranging from 20 to 100 °C (Fig. 2a). The OAT for SOD_*Ss*_ was 50 °C, which is close to that of other thermophile-derived SODs (50–70 °C). When added with NTD, rSOD_*Ss*_ exhibited optimal activity at 60 °C, similar to SODs (50–70 °C) derived from thermophilic bacteria such as *Thermoascus aurantiacus* var. *levisporus* (Song et al. 2009) and *Bacillus stearothermophilus* (Gligic et al. 2000), although lower than those (85–95 °C) reported from the hyperthermophilic archaea such as *Aquifex pyrophilus* (Yamano et al. 1999) and *Pyrobaculum aerophilum* (Whittaker and Whittaker 2000). The rSOD_*Ss*_ retained 74 % of its maximum activity even at 100 °C (compared to 64 % for SOD_*Ss*_). Although the relative activities were used for the comparison of the thermophilicities of the two SODs, the real activities of them are quite different. The initial enzymatic activities of SOD_*Ss*_ and rSOD_*Ss*_ investigated at 20 °C are 480 and 766 U mg^−1^, whereas the maximum at their individual OATs rise to 563 and 1152 U mg^−1^, respectively. Clearly, the NTD-fused rSOD_*Ss*_ are considerably more thermophilic than its counterpart without the NTD.

To investigate the effect of pH on SOD activity, the reaction was performed in buffers monitored at different pH from 3.0 to 10.0. As shown in Fig. 2b, both SOD_*Ss*_ and NTD-fused rSOD_*Ss*_ showed almost the same trends of activities under different pH conditions. The maximum activity of the wild type and recombinant SOD was observed at the slightly acidic pH 6.0. Outside their optimum pH ranges, the activities of both enzymes decreased quickly, suggesting that the pH preference of rSOD_*Ss*_ was not affected by fusion to the NTD.

In addition, the *T*_eq_ values of the SOD_*Ss*_ and rSOD_*Ss*_ were calculated using an equilibrium model to be 65.8 and 76.7 °C, respectively (Table 1, Additional file 1: Fig S2). The results indicated that the NTD also increased the optimum working temperature range of SOD_*Ss*_ with broader applicable potential.

**Table 1:** The equilibrium model parameters for SOD_*Ss*_ and rSOD_*Ss*_

Enzyme	Δ*G*_cat_^*‡*a^ (kJ mol^−1^)	Δ*G*_inact_^‡b^ (kJ mol^−1^)	Δ*H*_eq_^c^ (kJ mol^−1^)	*T*_eq_^d^ (°C)
SOD_*Ss*_	67.2	119.1	83.2	65.8
rSOD_*Ss*_	67.1	183.9	112.7	76.7

^a^Gibbs’ free energy of activation for an enzyme-catalyzed reaction

^b^Gibbs’ free energy of activation for the irreversible thermal inactivation of an enzyme

^c^Change in enthalpy for the E_act_ to E_inact_ transition

^d^The temperature at which the E_act_–E_inact_ equilibrium is at its midpoint

### The NTD enhances the thermostability and pH stability of SOD_*Ss*_

An OAT assay demonstrated that rSOD_*Ss*_ showed elevated thermophilicity after fusion to the NTD at its N-terminus. Therefore, we further examined the role of the NTD in rSOD_*Ss*_ thermostability and pH stability.

**Fig. 3:**
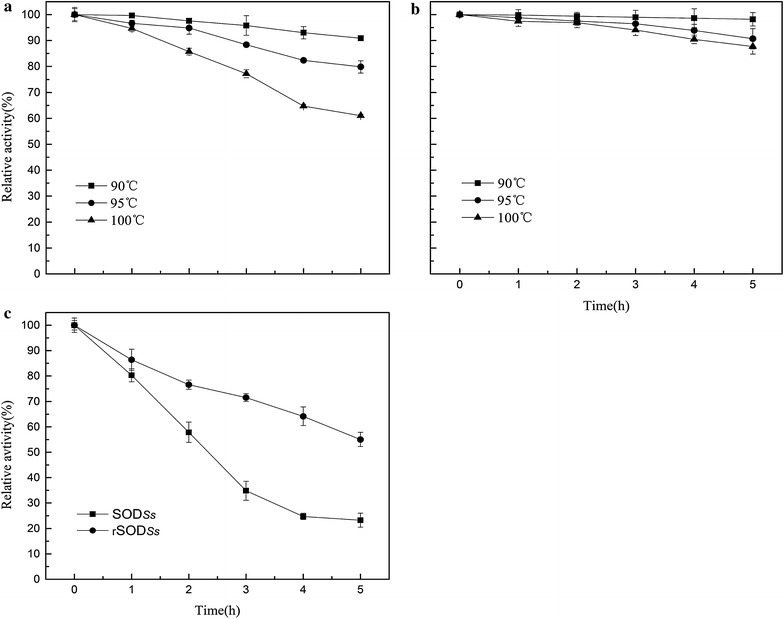
The thermostability of purified SOD_*Ss*_ (**a**) and rSOD_*Ss*_ (**b**) at 90–100 °C and the thermostability of SOD_*Ss*_ and rSOD_*Ss*_ at 105 °C (**c**). The enzymes were pre-incubated at various temperatures (90–105 °C, in increments of 5 °C), and aliquots were periodically withdrawn to test for residual activity using the standard assay described in the “Methods”. The activity of unheated SOD was defined as 100 %. Each point represents the mean (n = 3) ± the standard deviation

For thermostability test, the enzyme was pre-incubation at various temperatures (90, 95, 100 and 105 °C), and aliquots were withdrawn for intervals to test the residual activities. As shown in Fig. 3a, b, the activity of native SOD_*Ss*_ was slightly decreased when heating at 90 °C or above, with 40 % lost after incubation at 100 °C for 5 h. In contrast, the recombinant rSOD_*Ss*_ exhibited excellent thermostability over a range of temperatures from 90 to 100 °C, and it still retained 87 % of its activity after incubation at 100 °C for 5 h. Interestingly, dramatic difference on thermostability performance of these two enzymes was highlighted at extremely high temperature (105 °C, Fig. 3c). The half-life of rSOD_*Ss*_ activity at 105 °C was extrapolated to be 5.7 h, which was significantly longer than that of SOD_*Ss*_ (2.1 h). In addition, the deactivation energy of rSOD_*Ss*_ is higher than that of SOD_*Ss*_; they were estimated to be 246.7 and 215.3 kJ mol^−1^, respectively (Table 2). All the results suggested that the fused NTD had further enhanced the thermostability of SOD_*Ss*_.

**Table 2:** Thermodynamic parameters of SOD_*Ss*_ and rSOD_*Ss*_

Enzymes	*T* (°C)	*k*_*d*_ × 10^−3a^ (min^−1^)	*D* ^b^ (h)	*t*_1/2_^c^ (h)	*E*_*d*_^d^ (kJ mol^−1^)
SOD_*Ss*_	90	0.3	127.9	38.5	215.3
	95	0.7	54.8	16.5	
	100	1.6	23.9	7.2	
	105	5.3	7.2	2.1	
rSOD_*Ss*_	90	0.06	639.6	192.5	246.7
	95	0.3	127.9	38.5	
	100	0.4	95.9	28.8	
	105	2.0	19.1	5.7	

^a^ *k*_*d*_ is the deactivation rate constant (min^−1^)

^b^Decimal reduction time (*D*) is defined by Belitz and Gosch as the holding time required to reduce the enzymatic activity by one order of magnitude

^c^ *t*_1/2_ is the half-life time

^d^ *E*_*d*_ is the deactivation energy required to inactive the enzyme during a thermal inactivation process

**Fig. 4:**
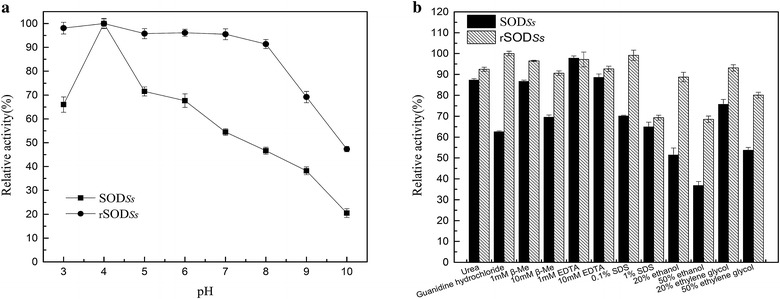
The pH stability of SOD_*Ss*_ and rSOD*Ss* (**a**). The residual SOD activity was evaluated after incubation at different pH values at 25 °C for 90 min and calculated as the percentage of the maximum activity. The buffer systems used were 50 mM sodium citrate (pH 3.0–8.0), Tris–HCl (pH 8.0 and 9.0), or glycine-NaOH (pH 9.0 and 10.0). Effects of inhibitors, detergents, and denaturants on SOD activity (**b**). Each enzyme was incubated with each inhibitor, detergent, denaturant and organic medium at various final concentrations in 50 mM sodium phosphate buffer (pH 7.8) at 25 °C for 30 min. The reaction mixture without inhibitor, detergent, denaturant or organic medium was used as a control and was defined as 100 %. The residual activities were measured by the standard assay as described in the “Methods”

pH stability test was performed by evaluating the residual activities of enzymes after incubation at different pHs from 3 to 10. Though with the same optimum pH, the rSOD_*Ss*_ showed remarkable stability (retaining >90 % of its initial activity) across a wide range of pH from 3 to 8, whereas SOD_*Ss*_ was quite unstable across this pH range, retaining <70 % of its maximum activity above the pH value of 5. It indicated that the acerbic and alkalic tolerance range of SOD_*Ss*_ was also broadened when appended with NTD (Fig. 4a).

### The NTD enhances the stress tolerance of SOD_*Ss*_

To evaluate the potential applications of SOD_*Ss*_ and rSOD_*Ss*_ in the industry, we examined the effects of stress and organic mediums on their enzyme activities. The effects of various inhibitors, detergents, denaturants and organic mediums on SOD activity were examined using EDTA, β-ME, SDS, urea, guanidine hydrochloride, ethanol and ethylene glycol (Fig. 4b). rSOD_*Ss*_ was considerably more resistant to these stresses than its counterpart lacking the NTD. When tested with guanidine hydrochloride at a final concentration of 2.5 M, SOD_*Ss*_ retained only 62 % activity, whereas the initial activity of the rSOD_*Ss*_ fused with NTD was not affected. Additionally, rSOD_*Ss*_ maintained 99 % of its initial activity after the addition of 0.1 % SDS, whereas SOD_*Ss*_ retained only 70 % of activity. The NTD also contributed to the organic medium tolerance of rSOD_*Ss*_, elevating 20–40 % of residual activity than that of SOD_*Ss*_ when subjected proteins to different concentration of ethanol and ethylene glycol (Additional file 1: Table S2).

### The NTD slightly alters the oligomerization state and composition of SOD_*Ss*_

**Fig. 5:**
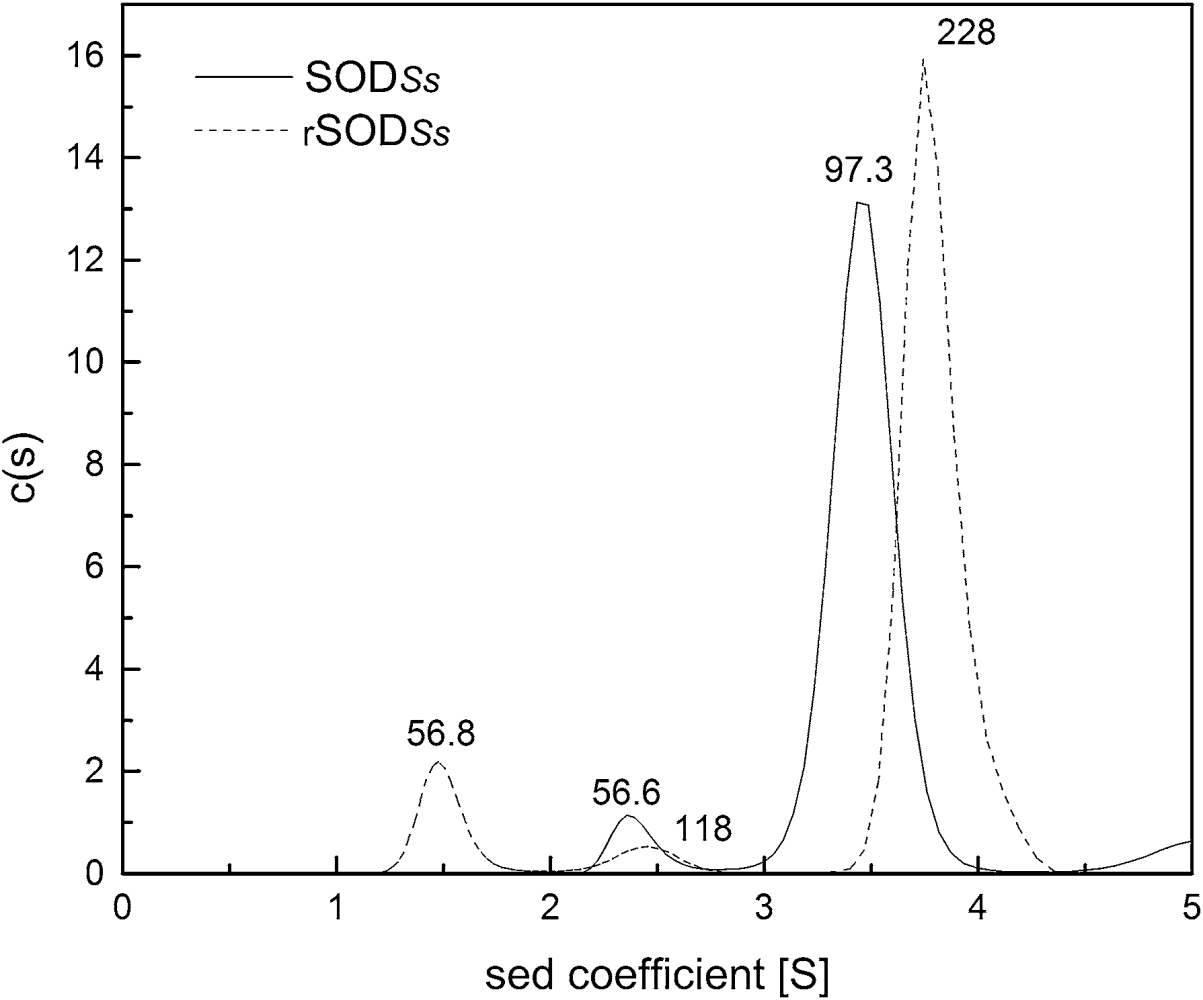
Analysis of the oligomerization states of SOD_*Ss*_ and rSOD_*Ss*_. The sizes of SOD_*Ss*_ and rSOD_*Ss*_ were determined by analytical ultracentrifugation. The estimated molecular masses (kDa) are provided above the peaks

Oligomerization has been proposed to contribute critically to the stability of proteins, and the stability of the quaternary structure is extremely important for the hyperthermostability of archaeal proteins. Analytical ultracentrifugation of SOD_*Ss*_ and rSOD_*Ss*_ yielded major peaks with sedimentation coefficients of 3.4 and 3.7 S, respectively (Fig. 5), corresponding to molecular masses of 97 and 228 kDa, respectively. This result indicates that both SOD_*Ss*_ and the NTD-fused rSOD_*Ss*_ exist primarily in a tetrameric form. In addition to small quantities of dimers present in both proteins, a small amount of rSOD_*Ss*_ existed as monomers. These results suggest that modification with the NTD results in insignificant alterations to the oligomerization state of SOD_*Ss*_.

## Discussion

Nowadays SODs have attracted tremendous attention and are widely used in the pharmaceutical, cosmetic, food, agriculture and environmental protection industries due to their excellent antioxidant properties (Angelova et al. 2001; Cullen et al. 2003; Emerit et al. 2006; Luisa Corvo et al. 2002; Melov et al. 2000; Yunoki et al. 2003). Most industrial SODs are obtained from naturally thermophilic or hyperthermophilic microorganisms, since increasing attention has been paid to improving the catalytic performance of enzymes under extreme but application-relevant conditions, such as high temperature, strong acid and alkali, or in organic and denaturing media (Kazlauskas and Bornscheuer 2009). Enzymes isolated from thermophilic (50–80 °C) or hyperthermophilic (>80 °C) microorganisms are usually more thermostable and more resistant to enzyme inhibitors, protein detergents, pH, and other denaturing agents than those from mesophilic (25–50 °C) or psychrophilic (<25 °C) microorganisms (Vieille and Zeikus 2001). The present study, which provides a new, convenient and universally applicable method, characterizes a recombinant SOD that was constructed by fusing the active sequence of thermophilic SOD_*Ss*_ with the NTD of SOD_*NG2215*_. The resulting enzyme, rSOD_*Ss*_, exhibited markedly improved thermophilicity, enhanced thermostability, stability over a wider pH range, greater stress resistance and organic medium tolerance than those of SOD_*Ss*_ without alterations in its optimum pH or oligomerization state. Notably, NTD fusion also increased the *T*_eq_ value of rSOD_*Ss*_, which is an indicator of the estimated optimal working temperature range. For industrial applications, the working temperature range of an engineering enzyme is of vital importance. The equilibrium model suggests that an increase in thermostability or thermophilicity alone will not necessarily result in improved activity at high temperatures unless the *T*_eq_ value is also increased (Eisenthal et al. 2006). Therefore, rSOD_*Ss*_ possesses comprehensively improved qualities and has considerable potential for related applications.

Protein engineering has emerged as an important tool to alter enzymes and the common strategies include site-directed mutagenesis and directed evolution (Bottcher and Bornscheuer 2010). Site-directed mutagenesis has been used for improving the thermostability of the thermostable Fe-SOD from *A. pyrophilus* (Lim et al. 2001) and a Cu/Zn-SOD from a polyextremophile higher plant, *Potentilla atrosanguinea* Lodd. var. *argyrophylla* (Kumar et al. 2012). However, site-directed mutagenesis requires a clear insight into the relationship between protein structure and function, and directed evolution requires a straightforward and efficient high-throughput screening method (Hong et al. 2007; Yang et al. 2012a, b). The oligopeptide fusion strategy has also been used to simultaneously improve the catalytic efficiency, thermostability and resistance to oxidation of an alkaline α-amylase (Yang et al. 2013). Though this method could be implemented without structural information or an efficient high-throughput screening method, it may be not suitable for all microbial enzymes, and the selection of oligopeptides will need to be tailored to each enzyme. In addition, enzyme immobilization has been applied to the thermostable Mn-SOD of *T. thermophiles* (Song et al. 2012). However, its applications are limited by SOD leakage and desorption. The subunits that constitute Fe- and Mn-SOD dimers or tetramers share a wide range of sequence similarities (which can be as low as 25.4 %) but possess virtually identical protein folds and active-site geometries (Jackson and Brunold 2004; Wintjens et al. 2004; Ding et al. 2012). SODA_*NG2215*_ and SODA_*Ss*_ share highly similar backbones, conserved metal-binding residues and nearly identical tetrameric structures (Additional file 1: Fig S1). Our previous work on mesophilic SODs (66 % identity with SODA_*NG2215*_) fused to a SOD_*NG2215*_ NTD, together with the present work (41 % identity with SODA_*NG2215*_) indicate that the NTD acts on their similar backbones to improve catalytic performance. Previous studies have shown that the structures of SODAs from mesophilic and thermophilic SODs are approximately identical (Wang et al. 2014b). We therefore propose that the drastic sequence alterations, not the few structural changes, contribute to the hyperthermophilicity of Fe- or Mn-SODs. The NTD used in the present work is suitable for all microbial Fe- or Mn-SODs, and it provides a universal and convenient way to generate more stable and tolerant SOD enzymes from both mesophilic and hyperthermophilic bacterial and archaeal enzymes.

The factors that contribute to the thermostability of proteins are numerous and complex; they include hydrogen bonding, ion-pair networks, hydrophobicity, molecular weight, hydrophobic interactions and secondary structures (Lim et al. 1997; Dello Russo et al. 1997; Wang et al. 2008a, 2009, 2014a; Yu et al. 2004; Hunter et al. 2002). Some proteins have even evolved more than one strategy to maintain their thermal tolerance. Structural analysis of SOD_*Ss*_ has revealed that it contains dominant inter-subunit ion-pairs along with high average of both hydrophobicity and amino acid weight, which contribute to heat tolerance (Dello Russo et al. 1997; Ursby et al. 1999). The rSOD_*Ss*_ enzyme exhibited superior thermophilicity and stress tolerance, suggesting that the extended NTD play a role synergistically with other thermophilicity-enhancement mechanisms. The extended NTD of one monomer may form special structures and connect with the NTDs from other subunits, thus promoting tetramer formation. However, the NTD fusion did not alter SODA backbone or the oligomerization state of SOD_*Ss*_, which further support our previous hypothesis that the NTD may provide an outer envelope that covers the temperature-sensitive hydrophobic residues or cavities on the surface of the active SOD ‘core’ and hence improves the formation of hydrogen bonds or polar interactions between the monomers without affecting interactions in the inner SOD ‘core’, which contributes the metal binding site and is important for tetramer formation (Wang et al. 2014b).

## Conclusions

In this study, we recombined the NTD to the N-terminal of SOD_*Ss*_ to further modify natively thermostable SOD. The biochemical properties (e.g. optimum temperature and pH, thermal stability, acidic and alkaline stability,stress stability) of the fusion protein (rSOD_*Ss*_) were characterized and compared with those of SOD_*Ss*_. In addition, the possible mechanisms responsible for improvement in enzyme stability were explored through analysis of oligomerisation state and comparison of structural modelling. The work presented here may provide a general and feasible strategy to enhance the thermophilicity and tolerance of both mesophilic and thermophilic Fe- or Mn-SODs from either bacteria or archaea.

## Additional file

10.1186/s40064-016-1854-9 Primers used for the construction of rSODSs in this study; **Table S2.** Effects of inhibitors, detergents, denaturants and organic medium on the activities of SOD_*Ss*_ and rSOD_*Ss*_; **Fig S1.** Structures of tetrameric SODA_*NG2215*_ and SODA_*Ss*_, superposition of monomeric SODA_*NG2215*_ and SODA_*Ss*_, active sites of SODA_*NG2215*_ and SODA_*Ss*_; **Fig S2.** The 3D plots of SOD_*Ss*_ and rSOD_*Ss*_.
