# Supplementary material for: Improving the thermostability and stress tolerance of an archaeon hyperthermophilic superoxide dismutase by fusion with a unique N-terminal domain
Source: Springerplus. 2016 Mar 1;5:241. doi: 10.1186/s40064-016-1854-9 (PMC4771647; doi:10.1186/s40064-016-1854-9)
Supplement: Supplementary file 1 — 10.1186/s40064-016-1854-9 Primers used for the construction of rSODSs in this study; Table S2. Effects of inhibitors, detergents, denaturants and organic medium on the activities of SODSs and rSODSs; Fig S1. Structures of tetrameric SODANG2215 and SODASs, superposition of monomeric SODANG2215 and SODASs, active sites of SODANG2215 and SODASs; Fig S2. The 3D plots of SODSs and rSODSs. [file 40064_2016_1854_MOESM1_ESM.docx]

**Title:** Improving the Thermostability and Stress Tolerance of an Archaeon Hyperthermophilic Superoxide Dismutase by Fusion with a Unique N-terminal Domain

**Authors**

Mingchang Li^1^, Lin Zhu^1^, Wei Wang^1,2,#^

**Affiliations:**

**^1^** Key Laboratory of Molecular Microbiology and Technology, Ministry of Education, TEDA Institute of Biological Sciences and Biotechnology, Nankai University, 23 Hongda Street, TEDA, Tianjin 300457, PR China

**^2^** Tianjin Key Laboratory of Microbial Functional Genomics, TEDA, Tianjin 300457, PR China

# Correspondence and requests for materials should be addressed to W.W.

([nkweiwang@nankai.edu.cn](mailto:nkweiwang@nankai.edu.cn))

**Table S1. Primers used for the construction of rSOD*_Ss_* in this study**

| Gene | Forward/reverse primer pair (5’-3’) | Product (bp) |
| --- | --- | --- |
| *sod_GTNG_2215-N_* | CCGGAATTCATGGACGACCAAACGTTGTTTGCCC^a^ | 732 |
|  | TTTGGAGAGTCAT CGAAACCGCCCGT |  |
| *Sod_Ss-C_* | ACGGGCGGTTTCG ATGACTCTCCAAA | 636 |
|  | CCCAAGCTTTTACTTCGTTAAATATTTCTGTAAC^a^ |  |
| *rsod_Ss_* | CCG*GAATTC*ATGGACGACCAAACGTTGTTTGCCC^a^ | 1,368 |
|  | CCCAAGCTTTTACTTCGTTAAATATTTCTGTAAC^a^ |  |

^a^ restriction enzyme sites are underlined

**Table S2. Effects of inhibitors, detergents, denaturants and organic medium on the activities of SOD*_Ss_* and rSOD*_Ss_***

| Inhibitors, detergents,  and denaturants | Residual activity^a^ | |
| --- | --- | --- |
|  | **SOD*_Ap_*** | **rSOD*_Ap_*** |
| Control | 100±2.2 | 100±2.6 |
| 1 mM EDTA | 97.8±1.1 | 97.2±3.5 |
| 10 mM EDTA | 88.5±1.7 | 92.7±1.3 |
| 1 mM β-ME | 86.7±0.6 | 96.5±0.3 |
| 10 mM β-ME | 69.5±1.2 | 90.6±1.1 |
| 0.1% SDS | 70.1±0.5 | 99.1±2.5 |
| 1% SDS | 64.8±2.3 | 69.4±1.1 |
| 2.5 M Urea | 87.2±0.7 | 92.5±0.9 |
| 2.5 M Guanidine hydrochloride | 62.5±0.5 | 100.0±1.1 |
| 20% ethanol | 51.4±3.3 | 88.8±2.3 |
| 50% ethanol | 36.8±1.9 | 68.5±1.5 |
| 20% ethylene glycol | 75.7±2.4 | 93.1±1.5 |
| 50% ethylene glycol | 53.6±1.4 | 80.1±1.4 |

^a^ The reaction mixture without inhibitor, detergent, or denaturant was used as a control and defined as 100%. The values represent the mean (n=3) ± the standard deviation of 3 replicates.


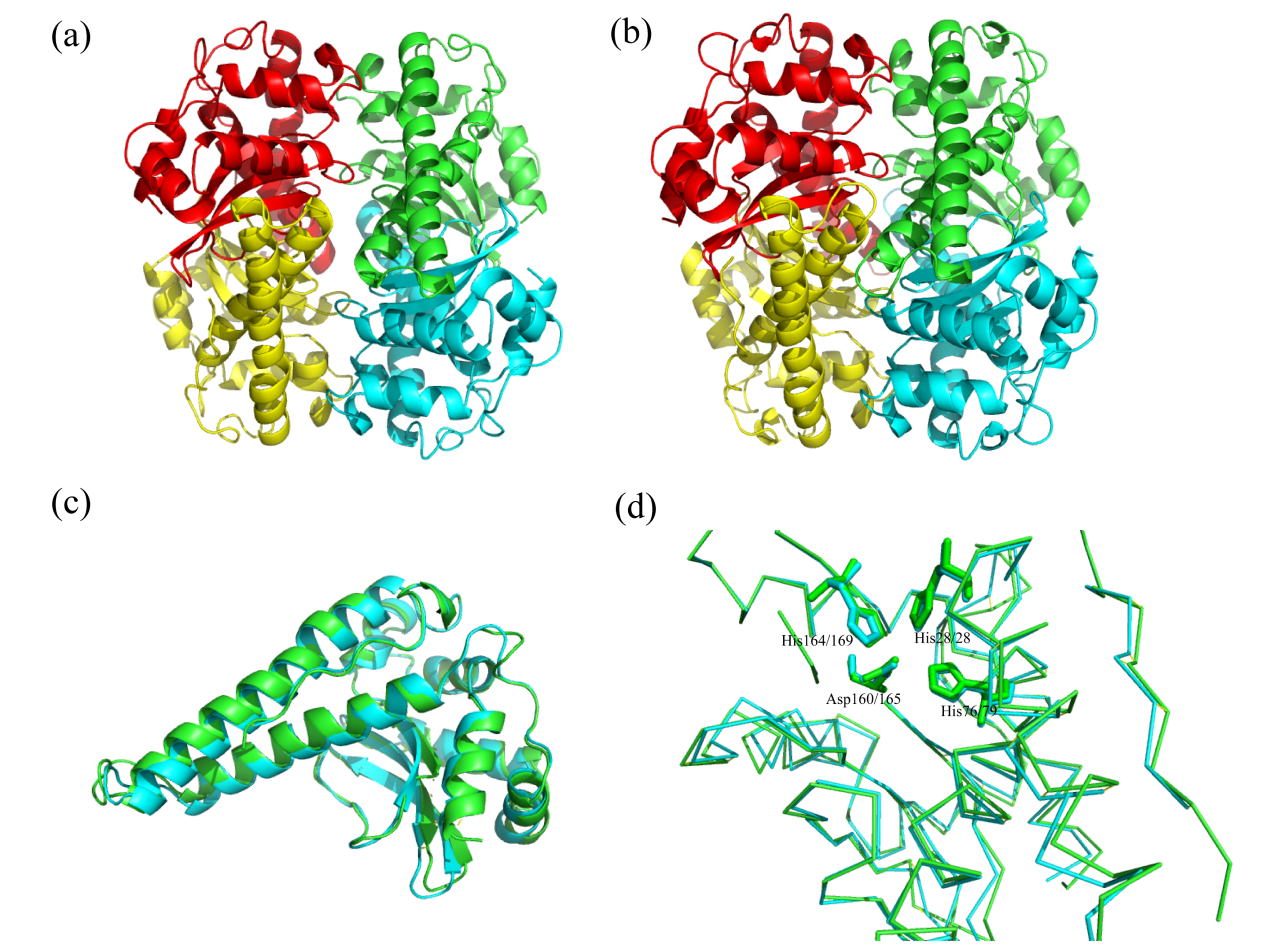


Fig S1. Structures of tetrameric SODA*_NG2215_* (a) and SODA*_Ss_* (b), superposition of monomeric SODA*_NG2215_* and SODA*_Ss_* (c), active sites of SODA*_NG2215_* and SODA*_Ss_* (d). SODA*_NG2215_* and SODA*_Ss_* are shown in blue and green, respectively. The structure of SODA*_NG2215_* was constructed using a Fe/Mn-SOD (3EVK) from *Pyrobaculum aerophilum* as template. The catalytic residues are labelled in blue (His28, His76, Asp160, and His164). The structure of SODA*_Ss_* was derived from the crystal structure of SOD*_Ss_* (1WB8). The catalytic residues are labelled in green (His28, His79, Asp165, and His169). The structure of SODA*_NG2215_* demonstrated good superposition with Fe-SODA*_Ss_* (Z-score, -1.28; root-mean-square deviation, 0.649 Å for 149 residues).


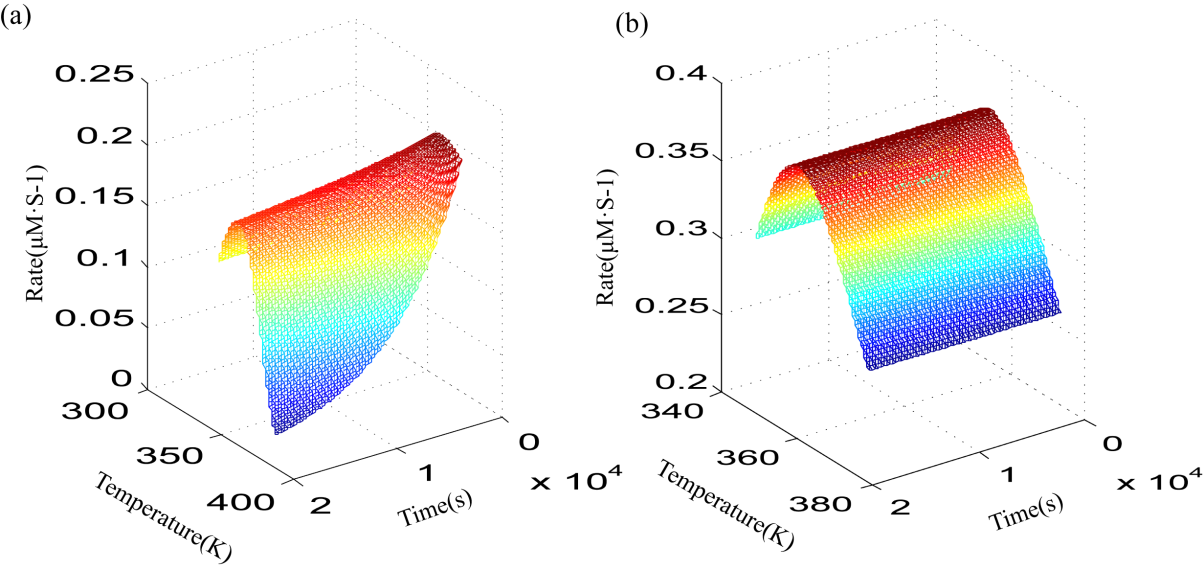


Fig S2. The 3D plots of SOD*_Ss_* (a) and rSOD*_Ss_* (b) were derived from the equilibrium model and were generated with Matlab version 7.
